# The German ‘Dunkelfeld’ Approach: When the Dark Figure of Sexual Delinquency Against Minors Remains Shady

**DOI:** 10.1007/s10935-025-00852-z

**Published:** 2025-04-25

**Authors:** Andrej König

**Affiliations:** Applied Social Sciences, University of Applied Sciences and Arts Dortmund, Emil-Figge-Straße 44, 44227 Dortmund, Germany

Since 2005, the slogan ‘Kein Täter werden’ (‘Don’t become an offender’) has been used in German media to promote confidential and anonymous treatment for pedohebephilic help-seekers to prevent sexual violence against minors. Over time, a comprehensive prevention network has been established, currently consisting of 13 treatment facilities across Germany. In the context of the ‘Kein Täter werden’ program, the term ‘Dunkelfeld’ refers to cases that remain outside the scope of law enforcement; individuals who are on probation or under ongoing legal investigation are not eligible for treatment. However, self-reported past convictions or undetected acts of sexual violence against minors are not grounds for exclusion.

Beier et al. (2024) present follow-up data on potential dynamic risk factors and self-reported sexual delinquency for *N* = 56 participants. These data were previously published by Nentzl and Scherner (2021). In addition, Beier et al. (2024) make several assertions that are inconsistent with criminological evidence and their earlier findings (Beier et al., 2015).

For instance, Beier et al. (2024) claim that their ‘Dunkelfeld’ approach addresses dynamic risk factors — pedophilia, cognitive victim empathy deficits, offense-supportive attitudes, and quality of life — associated with sexual offenses against minors and emphasize the need for well-funded aftercare to sustain long-term treatment effects. However, the authors do not provide any evidence to support the purported association between these dynamic risk factors and self-reported sexual delinquent behavior, some of which may fall below the legal threshold for prosecution. In contrast, criminological research on legally prosecuted sexual offenders has found no association between the suggested dynamic risk factors and criminal sexual recidivism (e.g., Blais et al., 2024).

Additionally, Beier et al. (2024) argue, citing results from von Franqué et al. (2023), that actuarial risk assessment tools are not applicable within the ‘Dunkelfeld’. Based on actuarial risk assessment tools von Franqué et al. (2023) report a low to medium risk of criminal sexual offenses in their sample of voluntary help-seekers (*N* = 165) from the ‘Kein Täter werden’ program. However, they found that actuarial risk assessment tools failed to predict self-reported sexual delinquent behavior against minors. This result is unsurprising, as these tools were designed to predict criminal convictions for sexual offenses, not self-reported delinquent behavior. Consistent with the low-risk profile of voluntary pedohebephilic help-seekers, von Franqué et al. (2023) report no cases of sexual delinquent behavior leading to legal prosecution or conviction. Thus, the actuarial tools appear to accurately predict the risk of legally prosecuted criminal sexual offenses. Beier et al. (2024) also support this conclusion, noting that only one participant (1.8%; 95% CI 0–10; *N* = 56) admitted being on probation for sexual abuse of a minor during the follow-up period.

Surprisingly, Beier et al. (2024, p. 895) regard the fact that 0% represents the lowest possible rate of criminal sexual offending as ‘cynical’ from a victim’s perspective, since 85.7% (*n* = 48; 95% CI 74–93) of their sample (*N* = 56) self-reported ongoing or first-time sexual delinquent behaviors during follow-up. This contradicts their own conclusion from a previous pilot study (Beier et al., 2015, p. 540):

*The significance of Dunkelfeld prevention and research activities with respect to sexual traumatization was again confirmed*,* despite the amount of persisting sexual offending as reflected in the official recidivism rates of 0%.*

The high rate of self-reported sexual delinquent behavior during treatment and follow-up raises concerns about potential iatrogenic effects of the ‘Dunkelfeld’ approach. According to a recent meta-analysis on indicators of treatment effectiveness (Holper et al., 2024), risk-focused treatment in low-risk sexual offenders has been shown to significantly increase sexual recidivism. This could explain the high rate of self-reported sexual delinquency observed in the follow-up study by Beier et al. (2024). However, without official criminal records — which are considered the gold standard for evaluating crime prevention effectiveness (Collaborative Outcome Data Committee, 2007) — and the use of controlled study designs, no causal conclusions can be drawn regarding the positive or adverse crime prevention effects of the ‘Dunkelfeld’ approach.

Sexual crimes against minors, prosecuted by law enforcement, place a significant burden on victims, society, and the judicial system. For two decades, the German ‘Dunkelfeld’ approach has positioned itself as a secondary crime prevention network, providing confidential and anonymous treatment to over 1,000 pedohebephilic help-seekers with the promise of preventing sexual offending. In Germany, confidentiality is an essential principle in clinical treatment settings and is protected by § 203 of the German Penal Code (StGB). According to § 34 StGB, confidentiality may only be breached by clinical treatment providers when there is an acute danger of serious sexual or non-sexual crimes. In addition to confidentiality, guaranteeing participants’ anonymity may increase the number of pedohebephilic individuals willing to contact the prevention network; however, it poses two key drawbacks: First, anonymity compromises cooperation with other healthcare providers, social services and law enforcement in cases of acute danger to potential victims of severe sexual violence. Second, it undermines a methodologically rigorous evaluation of the program’s crime prevention effectiveness, as official criminal records cannot be retrieved. Interestingly, a recent study from ‘Kein Täter werden’ in Switzerland reports that all of their pedohebephilic patients voluntarily disclosed their personal information during clinical assessment (de Tribolet-Hardy et al., 2024), which calls into question the necessity of guaranteeing participant anonymity.

Since the German ‘Dunkelfeld’ approach guarantees anonymity, self-reported sexual behavior — from those who are both willing and able to participate — remains the only available outcome measure of sexual offending. According to Beier et al. (2024, Fig. 1), this excludes 55.6% (*n* = 70) of the 126 help-seekers who completed treatment. Most were excluded due to missing contact information (*n* = 40), while fourteen explicitly refused or retracted participation. However, disengagement and general non-cooperation are known predictors of criminal sexual recidivism (e.g., Blais et al., 2024), which may introduce systematic biases in the evaluation of treatment outcomes. Moreover, sexual delinquent behaviors against minors are highly stigmatized, likely resulting in underreporting or minimization in self-reports. Due to these methodological limitations, meta-analyses on sexual offender treatment typically exclude primary studies that rely solely on self-reported delinquency (e.g., Holper et al., 2024). For the same reasons, established guidelines for evaluating sexual offender treatment (Collaborative Outcome Data Committee, 2007, pp. 64–70) regard self-reports and clinical records as invalid and unreliable outcome measures for sexual recidivism. To eliminate potential iatrogenic treatment effects — such as an increase in officially registered criminal sexual offenses — the ‘Dunkelfeld’ approach should adhere to established standards in prevention research (Gottfredson et al., 2015) and follow the guidelines for evaluating sexual offender treatment (Collaborative Outcome Data Committee, 2007). Certainly, participants’ self-reports of deviant sexual fantasies and behavior during treatment are important for assessing and managing individual risk. However, when self-reported sexual delinquency is the sole outcome measure, the ‘Dunkelfeld’ of sexual violence against minors will remain shady potentially harming victims, pedohebephilic help-seekers, and the German judicial system alike. In addition to the scientific and methodological concerns discussed, the flowchart in Beier et al. (2024, Fig. 1) appears to contain an error in the total sample size. Based on the diagram, the correct total should be *N* = 55, not *N* = 56.
